# Glaucoma care at ATBUTH Eye Clinic, Bauchi

**Published:** 2014

**Authors:** Abdull M Mahdi

**Affiliations:** Head of Department: Ophthalmology, Abubakar Tafawa Balewa University Teaching Hospital, Bauchi, Nigeria.

**Figure:**
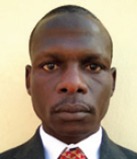
Abdull M Mahdi

The Abubakar Tafawa Balewa university teaching hospital (ATBUTH) in Bauchi, Nigeria, became a university teaching hospital for Bauchi State in 2010. Interest in glaucoma started in 2009, as many patients presented very late and were blind in one or both eyes.

The hospital has a large, mainly rural, catchment population. Most patients self-refer but referrals from district hospitals – which are staffed by ophthalmic nurses – are increasing. Patients with glaucoma now attend from across northern Nigeria.

Glaucoma services at the hospital is supported by the following six components of the local eye health system (refer also to the article on page 51).

## Leadership and governance

The clinic is headed by the author, who also sits on several hospital committees and boards and received specialised training in glaucoma in the UK in 2011. A senior ophthalmic nurse manages the day-to-day running of the clinic, e.g. stock control and ordering.

## Human resources for health

The department benefits from frequent locum consultant ophthalmologists, three optometrists, three resident doctors, two medical officers, eight ophthalmic nurses, two community health extension workers (who measure visual acuities), and five records staff. Residents visit to gain surgical experience. To reduce the load on the ophthalmologists, visual field assessment is undertaken by the optometrists, who are assisted by optometry interns.

Three nurses have been trained at the National Eye Centre in Kaduna to assist in theatre. Two doctors have been sent for ophthalmology residency training with plans to send two more. Weekly departmental meetings are held to discuss cases.

## Technology, equipment, infrastructure and medicines

The eye department has a reception area for records and fee payment, a large waiting area, space for measuring visual acuity, six offices for consultants, and additional consulting rooms for junior doctors, optometrists and nurses. There is also an operating theatre and a minor procedures room. There is a dedicated room for glaucoma diagnostic equipment, a glaucoma research project office, and a glaucoma counselling room where motivational interviewing (a type of supportive counselling) is provided by two inteviewers.

In 2004, the clinic had only one slit lamp, a Schiotz tonometer, a lens trial set and a loupe. For detection of glaucoma there are now 1-, 2- and 4-mirror gonio lenses, tonometers (Goldmann, airpuff and Perkins), lenses for retinal/disc examination, a stereoscopic digital fundus camera for optic disc imaging and a Twinfield visual field analyser. In 2010, the clinic purchased a diode laser for trans-scleral cycloablation.

The eye clinic stocks some glaucoma medication for patients.

## Health financing

The department is funded by the hospital, which runs a revolving fund. This fund works by giving some seed money to the clinic to purchase all the consumables and drugs needed to run the unit. As service is provided, this money is recovered from patients' fees and any profits are used to replenish the revolving fund.

There are three systems for payment: user fees, the National Health Insurance Scheme (NHIS) and retainerships. In retainerships, companies or organisations enter into an agreement with the hospital to treat their staff whenever they need medical attention. These organisations deposit money with the hospital, which is then used to offset any bills incurred by their employees.

There is a social welfare department to assist patients who are unable to pay for services. The hospital finances all staff training, including specialist training, and equipment for eye care is purchased by the hospital, mostly from profits from the revolving fund.

## Health information systems

The availability of new clinic space and staff allowed a more organised system of record keeping to start in 2010: new patients obtain a card to see the ophthalmic nurse or optometrist, and a folder is only opened if consultation with a doctor is required. The system is being made electronic, which is essential for monitoring glaucoma patients.

**Figure:**
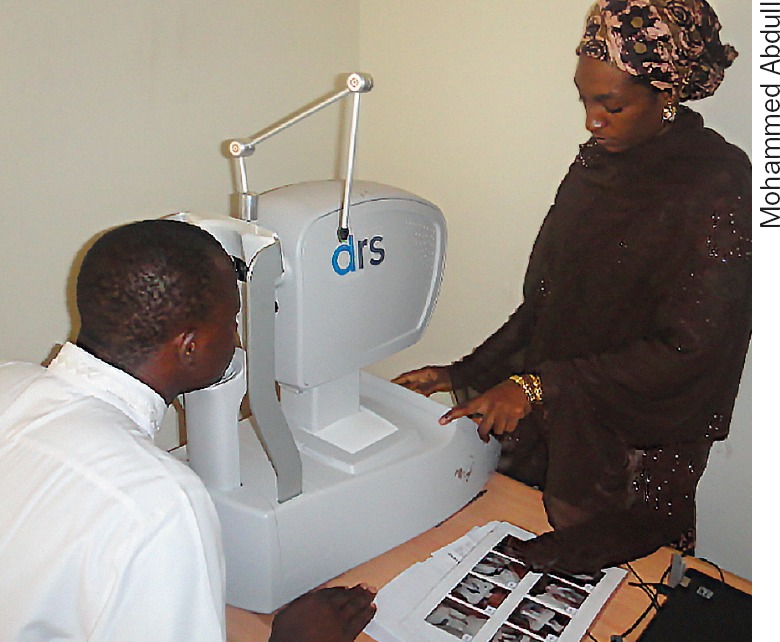
Digital fundus photography for glaucoma screening

## Service delivery

Optometry interns screen all patients aged ≥30 years who attend the clinic for glaucoma using optic disc assessment. All those with suspicious discs are examined in detail. In 2013, glaucoma was diagnosed in more than 500 patients.

Since laser treatment became available (which is explained to patients as computer light treatment), many patients have accepted laser rather than trabeculectomy. Over 300 patients have been treated with laser so far, 160 of whom are being closely followed up. Laser gives good lowering of IOP in the short term and results are being routinely recorded. These data will be published when 1-year follow-up data are available. In 2012, we started a clinical trial to assess the effectiveness of motivational interviewing to increase patients' uptake of laser or trabeculectomy when this is the treatment of choice.^1^

A health education pamphlet on glaucoma, suitable for those who are not literate, has been developed.

## Conclusion

The support of senior management in the central hospital has been very important in the development of the eye clinic. The commitment of senior management to the eye clinic is the result of several factors:

- eye clinic staff involvement in the management of the central hospital
- building good relationships with people in a position to support the clinic and increasing their awareness of glaucoma
- positive feed back from patients about the high quality care they have received
- prudent management of resources, including the revolving fund.

The clinic needs further strengthening as a tertiary glaucoma centre, including the purchasing of better equipment. Once this has been achieved, secondary level centres in the state will be supported by the clinic to improve their care and referral of glaucoma patients. Our long-term goal is to support early detection and referral of glaucoma at primary eye care level, with effective management of glaucoma at secondary and tertiary levels.

